# Mitochondrial (Dys) Function in Inflammaging: Do MitomiRs Influence the Energetic, Oxidative, and Inflammatory Status of Senescent Cells?

**DOI:** 10.1155/2017/2309034

**Published:** 2017-12-27

**Authors:** Angelica Giuliani, Francesco Prattichizzo, Luigina Micolucci, Antonio Ceriello, Antonio Domenico Procopio, Maria Rita Rippo

**Affiliations:** ^1^Department of Clinical and Molecular Sciences, DISCLIMO, Università Politecnica delle Marche, Ancona, Italy; ^2^IRCCS Multimedica, 20099 Sesto San Giovanni, Italy; ^3^Insititut d'Investigacions Biomèdiques August Pi i Sunyer (IDIBAPS), C/Rosselló 149-153, 08036 Barcelona, Spain; ^4^CIBER de Diabetes y Enfermedades Metabólicas Asociadas (CIBERDEM), Madrid, Spain; ^5^Center of Clinical Pathology and Innovative Therapy, Italian National Research Center on Aging (INRCA-IRCCS), Ancona, Italy

## Abstract

A relevant feature of aging is chronic low-grade inflammation, termed inflammaging, a key process promoting the development of all major age-related diseases. Senescent cells can acquire the senescence-associated (SA) secretory phenotype (SASP), characterized by the secretion of proinflammatory factors fuelling inflammaging. Cellular senescence is also accompanied by a deep reshaping of microRNA expression and by the modulation of mitochondria activity, both master regulators of the SASP. Here, we synthesize novel findings regarding the role of mitochondria in the SASP and in the inflammaging process and propose a network linking nuclear-encoded SA-miRNAs to mitochondrial gene regulation and function in aging cells. In this conceptual structure, SA-miRNAs can translocate to mitochondria (SA-mitomiRs) and may affect the energetic, oxidative, and inflammatory status of senescent cells. We discuss the potential role of several of SA-mitomiRs (i.e., let-7b, miR-1, miR-130a-3p, miR-133a, miR-146a-5p, miR-181c-5p, and miR-378-5p), using miR-146a as a proof-of-principle model. Finally, we propose a comprehensive, metabolic, and epigenetic view of the senescence process, in order to amplify the range of possible approaches to target inflammaging, with the ultimate goal of decelerating the aging rate, postponing or blunting the development of age-related diseases.

## 1. Introduction

Aging constitutes the major risk factor for a variety of diseases with a high-incidence in the Western society. Type 2 diabetes (T2DM), metabolic syndrome, cardiovascular diseases (CVD), neurodegenerative diseases, and a plethora of common cancer types share aging as a common risk factor and therefore are often referred as aging-related diseases (ARDs). A pervasive feature of aging is a progressive and chronic state of systemic low-grade inflammation referred as inflammaging [1]. Major ARDs all share a common inflammatory background. The role of various inflammatory molecules—mainly tumor necrosis factor- (TNF-) *α*, interleukin- (IL-) 1b, IL-6, and transforming growth factor- (TGF-) *β*—in the promotion or exacerbation of a wide range of ARDs is increasingly emerging [1]. A number of mechanisms, pathways, and cell types have been shown to possibly contribute to inflammaging [2]. Initial hypothesis affirmed that inflammaging was mainly due to the long-lasting exposure to acute and chronic infections and the consequent life-long antigenic burden [3]. However, a long list of sterile potential sources of inflammatory molecules during both the cellular and the organismal aging process has been proposed [2].

The “old” free radical theory of aging (FRTA) states that organisms age because cells accumulate free radical-induced damage over time [4]. One interesting view that coupled FRTA with inflammaging is the oxi-inflammaging theory [5]. Accordingly, excessive or uncontrolled free radical production can induce an inflammatory response, and free radicals are themselves inflammation effectors. Oxidative metabolism at mitochondrial level is probably the major source of intracellular ROS production, which in turn represents a life-long-lasting stress. Aging-related ROS accumulation can contribute to telomere attrition [6], oxidative genomic damage [7], but can even act as signaling molecules in the development and maintenance of the senescent phenotype of the cell [8].

However, increasing evidence suggests oxidative stress is not per se a major determinant of aging; in fact, (i) deletion of many antioxidant enzymes increases rather than decreases life span and health span in various models of lower organisms and (ii) interventions with nonspecific antioxidant molecules do not reduce the incidence of ARDs [9–12]. On the contrary, (excessive) inflammation appears as a phenomenon that alone is sufficient to reduce both life span and fitness at old age [13, 14]. Thus, in aging cells, mitochondria-derived ROS and oxidative stress should be regarded for their role as proinflammatory triggers rather than damaging molecules that progressively disrupt cell components and cellular homeostasis alone.

MicroRNAs (miRNAs o miRs) are small noncoding RNAs involved in gene expression modulation, primarily silencing the mRNA target by binding its 3′-untranslated region (UTR) in the cytoplasm. However, increasing evidence proves that miRNAs can also exert posttranscriptional control when bound to a region outside of the 3′UTR and specifically within the 5′UTR and coding regions of the mRNA target. Moreover, in some cases, they activate transcription of a specific gene or stabilize the mRNA [15]. A single miRNA has the ability to regulate multiple targets and, in turn, a single mRNA can be targeted by several miRs [16]. Because of their regulatory functions, these small single strand RNAs are virtually implicated in all cellular processes. Over the years, a set of miRNAs with a well-recognized role in inflammaging, organismal aging, and development of ARDs has been defined [17–19]; examples are miR-146a-5p, miR-21, miR-126a-3p. Interestingly, recent studies suggest that a subset of these microRNAs can be found inside or indirectly affect the mitochondria (mitomiRs) [20]. They all have a nuclear encoding, but the existence of few small noncoding RNAs of mitochondrial origin is documented [21].

In this review, we explore the role of mitochondria in fostering “inappropriate” inflammatory responses during the aging process. We take advantage of cellular senescence as a fractal model to delineate the contribution of dysfunctional mitochondria to the inflammaging process since recent discoveries indicate that mitochondrial activities sustain or drive the inflammatory program of senescent cells. In addition, we explore mitochondria-linked mechanisms that can promote inflammaging independently of senescent cells. Finally, we explain the hypothesis that senescence-deregulated mitomiRs could directly affect mitochondrial function by targeting mtDNA, thus influencing the energetic, oxidative, and, in turn, inflammatory status of senescent cells, possibly playing a role in organismal aging.

## 2. Senescent Cells Fuel Inflammaging through the Senescence-Associated Secretory Phenotype

Recently, cellular senescence has been suggested as a relevant contributor to both the inflammaging and the aging process. This statement is mainly sustained by data on animal models showing that periodic clearance of senescent cells is accompanied by a mean life span and health span extension, coupled with a reduced inflammatory gene expression in multiple tissues, including kidney and the heart [22]. Senescent cells are characterized by a permanent cell cycle arrest, accompanied by morphological and gene expression changes [23]. They are usually identified by the high expression of proteins promoting cell cycle arrest, that is, p16, p21, and p53, the expression of DNA damage markers, that is, *γ*H2AX phosphorylation, senescence-associated heterochromatin foci (SAHF), and telomere-associated DNA damage foci (TAF), and an increased activity of senescence-associated (SA) beta-galactosidase (*β*-Gal) [24]. Many stimuli can foster the acquisition of the senescent phenotype, for example, radiation, telomere erosion or damage, oncogenic stress, oxidative stress, and a number of DNA damaging agents [24–26]. In turn, senescent cells can acquire a proinflammatory phenotype named senescence-associated secretory phenotype (SASP). SASP consists in secretion of inflammatory cytokines, growth factors, and proteases [24]. Increased levels of SASP-related compounds have been reported in a number of human ARDs such as diabetes [27–29], atherosclerosis [30], and cancer [31]. More importantly, senescent cell accumulation in selected microenvironments—with a subsequent locally increased concentration of inflammatory cytokines—drives the pathogenesis of prototypical ARDs, that is, osteoarthritis and osteoporosis [32, 33].

A plethora of pathways and proteins has been implicated with inflammatory molecule secretion in senescent cells, for example, NF-*κ*B, mTOR, and JAK [27, 34]. The classical SASP network relies on cell surface IL-1*α* as an essential cell-autonomous regulator of IL-6/IL-8 secretion [35]. However, recent findings indicate that an alternative inflammatory network is possible in senescent cells and that it is strictly dependent on mitochondrial function [36]. In addition, mitochondria appear as indispensable for the proinflammatory and the proaging features of senescent cells [37].

## 3. Senescent Cells Bear a Distinct Metabolic Phenotype, and Mitochondrial Dysfunction Is Partly Responsible for Their Proinflammatory Program

The senescence process is accompanied by metabolic changes within the cell. Many of the proteins important for the senescence process have a pivotal metabolic function [38]. As a result, senescent cells show a distinct metabolic phenotype. They bear an active metabolic state, maybe due to the acquisition of their peculiar secretory profile, which implies a high transcriptional activity and possibly a high metabolic demand. Senescent cells increase their glucose consumption, a phenomenon not coupled by an increased energetic state. In fact, senescent cells present a strong reduction in the amount of ATP in favor of adenosine diphosphate (ADP) and adenosine monophosphate (AMP) [39]. The increases of AMP/ATP and ADP/ATP ratios are sensed by and activate AMP-activated protein kinase (AMPK). [40]. AMPK activation, in turn, can induce senescence via p53 phosphorylation [41], whose activation has been associated with the promotion of glycolysis and oxidative phosphorylation, but even to downmodulation of glycolysis [42]. Furthermore, mTOR, one of the master regulators of the SASP but even of the senescence process itself [43], is strictly linked to nutrient availability, in particular to amino acids and glucose [44]. The same nutrients promote the activation of the transcription factor NF-*κ*B, which plays a pivotal role in the inflammatory program of both immune cells and senescent cells. NF-*κ*B governs energy homeostasis and metabolic adaptation by controlling the balance between glycolysis and respiration for energy provision [45]. Inhibition of NF-*κ*B diminishes oxygen consumption and causes reprogramming to aerobic glycolysis (i.e., the Warburg effect) in mouse embryonic fibroblasts (MEFs) under basal culture conditions and induced necrosis on glucose starvation [45]. This NF-*κ*B-dependent modulation of oxidative phosphorylation system (OXPHOS) involves the p53-mediated upregulation of mitochondrial synthesis of cytochrome c oxidase 2 (SCO2) [46], a subunit of complex IV of the mitochondrial electron transport chain [46–48].

The OXPHOS is composed of complexes I-IV (the electron transport chain, ETC), located within the mitochondrial inner membrane, and generates a gradient of H^+^ ions, which drives the ADP phosphorylation via the ATP synthase (F_o_F_1_ ATPase—complex V) [49, 50]. During aerobic respiration, a variable percentage of electrons leaks from the ETC, particularly from complexes I and III, prematurely reduces oxygen, and generates ROS [51]. This process is exacerbated in senescent cells and leads to an overproduction of ROS.

In “usual” senescence models, mitochondria do play a fundamental role. In fact, the absence of mitochondria reduced a spectrum of senescence effectors and phenotypes while preserving ATP production via enhanced glycolysis. A plethora of senescent-associated changes was dependent on mitochondria, particularly the proinflammatory phenotype. A DNA damage response (DDR) pathway converging on mTORC1 phosphorylation promoted PGC-1*β*-dependent mitochondrial biogenesis, contributing to ROS-mediated activation of the DDR and cell cycle arrest. Of note, the reduction in mitochondrial content *in vivo*, by either mTORC1 inhibition or PGC-1*β* deletion, prevented senescence in the aging mouse liver [37].

A recent paper clearly shows that mitochondrial dysfunction per se is sufficient to trigger a particular form of senescence accompanied by a peculiar proinflammatory program but independent from ROS and DNA damage. In this model, mitochondrial dysfunction-associated senescence (MiDAS) lacks an IL-1/NF-*κ*B-dependent mechanism but would involve secretion of factors other than “classical” SASP factors. Major MiDAS-associated molecules are IL-10, TNF-*α*, and CCL27, which are secreted in a NF-*κ*B-independent manner. This novel senescence phenotype would result from a reduced NAD^+^/NADH ratio, which in turn may cause AMPK and p53 activation [36]. Of note, treatment with a NAD^+^ precursor delays senescence of neural and melanocyte stem cells, increasing mouse life span [52].

## 4. Mitochondrial Dysfunction and Damage Promote Inflammaging through ROS- and PRR-Mediated Mechanisms

Life-long stimulation of the cells of innate immunity is still one of the more consistent hypotheses regarding the cellular origin of inflammaging. Beyond bacteria- and virus-derived products, a long list of endogenous molecules can promote this inflammatory program [2]. When misplaced, many “normal” cell components are recognized as damage-associated molecular patterns (DAMPs), which are recognized by pattern recognition receptors (PRRs), a large family of proteins that include toll-like receptors (TLRs) and NOD-like receptors (NLRs). During aging, PRRs are subjected to a prolonged and increased exposure to “stress” molecules that leads to chronic inflammatory responses.

Cell injury due to various stressors can trigger the release outside of the mitochondrial matrix of mitochondrial DNA (mtDNA), *N*-formyl peptides and lipids, such as cardiolipin, that can act as DAMPs by activating receptors of the innate immunity [2, 53] and the inflammasomes. In particular, there is a direct link between released oxidized mtDNA, activation of NLRP3, the most studied NLR, and caspase-1-mediated maturation of IL-1*β* and IL-18 [54, 55]. Accordingly, increasing evidence suggests an important role of inflammasomes in major ARDs [27]. For instance, the ablation of Nlrp3 gene in mice is able to attenuate inflammaging, thus increasing health span [13]. Once activated, inflammasome promotes the transmission of the senescence bystander cells via the release of inflammatory mediators [56]. In turn, mitochondria-derived ROS can activate the inflammasome, probably through the Trx/TXNIP complex, further linking mitochondrial function to low-grade inflammation [57]. Accordingly, ROS themselves can partly mediate the senescence “bystander effect”, that is, the transition of the senescent phenotype from a SC to neighbor cells [58].

MtDNA can even bind TLR9, which senses DNA of bacterial and viral origin [59]. Because of the prokaryotic origin of mitochondria, mtDNA contains a significant number of unmethylated CpG DNA repeats, similarly to bacterial genomes. This is sufficient to trigger TLR9 activation, leading to NF-*κ*B signaling and increased expression of proinflammatory cytokines, such as TNF-*α*, IL-6, and IL-1*β* [60]. Further, mtDNA can even activate the cytosolic DNA sensor cyclic GMP-AMP synthase (cGAS) [61]. During an infection, DNA of microbial origin by binding to cGAS activates the cGAS/stimulator of interferon gene (STING) pathway thus triggering expression of inflammatory genes. Since DNA damage plays a prominent role in the development of the SASP [62], this pathway could be considered a mediator of inflammaging. Of note, even mitochondrial DNA (mtDNA) can bind the cytosolic cGAS, suggesting that mitochondria can play a major role in the senescence/SASP process in different manners [63, 64]. Notably, mtDNA can be released into extracellular fluids as a product of tissue damage and its plasma levels increase during aging and in selected ARDs [65], supporting the notion that circulating mtDNA is part of the systemic communicome initiating and propagating inflammaging [2].

Human mtDNA consists of circular, double-stranded, supercoiled molecules that are found in each cell in one up to 10,000 copies, depending on the bioenergetic requirements of the tissue and whose number declined with aging [66]. It encodes 13 subunits of respiratory chain complexes (in addition to 2 rRNAs and 22 tRNAs), which are transcribed and translated directly within the mitochondrion by its own gene expression system. These polypeptides include indispensable components of the complex I (7 subunits—ND1-ND6, ND4L), III (apocytochromeb), IV (3 subunits—COX I–III), and of F0/F1 ATP synthase (ATPase6 and ATPase8). The mitochondrial genome has a higher mutation rate than the nuclear genome; thus, its mutations accumulate during aging [67]. However, Greaves et al. have shown that selected pathogenic mutations increase and clonally expand during aging, but not the frequency of mutation [68]. Of note, mtDNA mutation accumulation in mouse tissues is influenced by the nuclear genetic background and correlates to neither cellular ROS content nor tissue senescence [69]. Interestingly, a number of genetic variants of the mtDNA, named haplogroups, have been identified. These, each containing functional single nucleotide polymorphism (SNPs), affect the oxidative metabolism of mitochondria, and several of them are associated to extreme longevity [70]. The interaction between nuclear genes and mtDNA has been proposed as a possible mechanism explaining a favorable/unfavorable inflammaging profile and consequently successful or unsuccessful aging [71]. Overall, these data suggest that any physiological SNP or pathogenic mutation in mtDNA could induce respiration alteration with a wide range of possible resulting (pathological) phenotype, affecting in turn inflammaging and ARD development [72, 73].

## 5. A Subgroup of MicroRNAs Targets mtDNA or Nuclear mRNA with Mitochondrial Function

The miRNA machinery is known to primarily act in the cytoplasm. miRNAs have also been detected in membrane-bound compartments, such as secreted vesicles and mitochondria [74]. Through different experimental approaches in different mammalian species, a number of studies have enabled identification of “signatures” of miRNAs located in the mitochondria. To date, they are summarized under a descriptive term: mitomiRs [75]. Indeed, this name includes nuclear-encoded miRNAs that translocate into the mitochondria organelle and target either mitochondrial or nuclear mRNA. Early studies on the subject are mostly descriptive to detect the presence of microRNAs within the mitochondrion. Kren and colleagues first detected 15 nuclear-encoded mitomiRs from rat livers that seemed to be involved in the modulation of genes associated with apoptosis, cell proliferation, and differentiation [76]. Bian et al. identified a pool of 20 miRs highly expressed in mitochondria of mice liver. Interestingly, mitochondria have a unique population of miRNAs, independent of the total cellular abundance of miRs and they may be involved in the regulation of mitochondria-specific and general cellular functions [77]. For the first time, in 2011, Barrey and coworkers showed the presence of pre-miRs inside mitochondria, postulating that some pre-miRNA sequences could be processed to mature miRNAs, which could be immediately active on the mitochondrial transcripts or exported in the cytosol in order to interfere with genomic mRNA [78]. Subsequently, other groups identified new mitomiRs from HeLa [79, 80], HEK293 [80], 143B cells [81], 206*ρ*° cells [82], and mouse heart [83] (Tables 1 and 2). The notion emerging is that mitochondria have a discrete and unique pool of mitomiRs; the association of miRs with mitochondria is species and cell type-specific [75].

## 6. MitomiRs Targeting Mitochondrial Genome Are Deregulated by Organismal Aging or Cellular Senescence

Mitochondria maintain and express their own genome that may be regulated by microRNAs. As mtDNA encodes for 13 subunits of the electron transport chain, their regulation by mitomiRs may have profound effects on ATP synthesis and ultimately may influence the whole mitochondrial function.

Bioinformatics analysis suggested that mitomiRs can target various mitochondrial transcripts: several potential mitochondrial targets for let-7b (ATP6, ATP8, COX2, and ND5) [78] as well as for miR-146a-5p and miR-181c-5p [82] have been described; miR-133a was predicted to target ND1 [78], whereas miR-130a-3p targets COX3 [76]. Using ingenuity pathway analysis (IPA) software to analyze mtDNA targets, Jagannathan et al. observed potential interactions between all 13 mitochondrial genome-encoded electron transport chain proteins with mitomiRs. The functional significance of a specific miRNA in heart-derived mitochondria was demonstrated for the first time by Das and colleagues: miR-181c-5p originates from the nuclear genome, is processed in the cytosol, and translates to the mitochondria, where it regulates mitochondrial energy metabolism by targeting mt-COX1 mRNA. Overexpression of miR-181c-5p results in a loss of mt-COX1 protein, resulting in an imbalance among the mitochondria-encoded subunits in complex IV, thus promoting ROS generation. Perturbations induced by miR-181c-5p could have important consequences in myocardial pathophysiology [84]. More recently, *in vivo* administration of miR-181c-5p in rats confirmed these data leading to complex IV dysfunction, altered mitochondrial metabolism, and ROS generation, ultimately promoting heart failure [85].

On the contrary, when miR-1 localizes into mitochondria, it promotes mitochondrial translation of ND1 and COX1 mtDNA-encoded transcripts while repressing its nuclear DNA-encoded targets in the cytoplasm. In this way, miR-1 enhances protein synthesis and ATP production, required for muscle cell differentiation [74]. This study suggests the possibility that the localization and relative abundance of a specific microRNA in the cytoplasm and mitochondria determines its different role in the regulation of specific targets and in the coordination of mitochondrial activities [86].

Another study showed that miR-1, together with other two mitomiRs, miR-133a and let-7b, is essential for adult skeletal muscle differentiation and maintenance; for this reason, they are called myomiRs [87, 88]. Muscles contain a high mitochondrial content, in order to provide massive demand of ATP for movement, postural maintenance, and respiration. During aging, skeletal muscle mitochondria revealed a progressive decline in respiratory chain function, a phenomenon associated with insulin resistance and type 2 diabetes mellitus [89]. These miRs seem to modulate some key proteins of OXPHOS complexes, so their deregulation may affect ETC functionality. Of note, some of these myomiRs are released in circulation after physical exercise [90]. Physical activity is associated to a plethora of beneficial effects and the underlying mechanisms are not fully understood. For instance, high-intensity aerobic interval exercise is associated with wide proteomic changes in the muscle of old subjects, with evident phenotypic gains in muscle mitochondrial function. Noticeable, mRNA expression changes do not overlap protein expression changes, suggesting that enhanced protein translation, but possibly even epigenetic mechanisms influence such positive adaptation [91].

Another remarkable study showed a redistribution of mitomiRs in the diabetic heart. Cardiac tissue is rich in mitochondria with spatially distinct subpopulations: subsarcolemmal mitochondria (SSM), located beneath the cell membrane, and interfibrillar mitochondria (IFM), situated between the myofibrils. MitomiRs were differentially regulated in the two mitochondrial subpopulations in diabetes relative to control. In addition, mitomiR-378-3p is highly expressed in diabetic IFM (versus IFM control) and targets mt-ATP6 with a concomitant reduction in the functionality of the ATP synthase*. In vivo*, miR-378-3p antagomir delivery led to the preservation of ATP6 protein levels in diabetic IFM, similar to nondiabetic control. Interestingly, miR-378-3p antagomir resulted in an increase of ATP synthase activity, which was significantly decreased in diabetic [83]. MiR-378-3p is located in the first intron of the peroxisome proliferator-activated receptor gamma (Ppargc1b) gene, which encodes for PGC-1*β*. PGC-1*β* is preferentially expressed in tissues with relatively high mitochondrial content, and miR-378-3p is coexpressed with its host gene and seems to counterbalance the metabolic actions of PGC-1*β*. Mice genetically lacking miR-378-3p exhibit enhanced mitochondrial fatty acid metabolism and elevated oxidative capacity of insulin-target tissues [92], proving its key role in mitochondrial respiration.

According to their role in age-related diseases, for example, heart failure and diabetes [18–20, 74, 85, 93], many mitomiRs have been described to be deregulated during organismal and cellular aging (SA-miRs). Thus, it has been hypothesized that SA-miRs and mtDNA-targeting mitomiRs play a major role in the mitochondrial dysfunction observed in both cellular senescence and the inflammaging process [94]. Notably, among all mitomiRs targeting mitochondrial genome, a subset of miRs has been associated to inflammatory processes, that is, miR-130a-3p [95] or to both inflammation and aging, that is, let-7b [96, 97], miR-146a-5p [98], miR-181c-5p [99, 100], miR-133a [101, 102], and miR-1 [103, 104].

MiR-146a-5p is one of the best-characterized miRNAs involved in both SASP and inflammaging. NF-*κ*B activation initiates the transcription of proinflammatory cytokines and of miR-146, which in turn directly targets IRAK1 and TRAF6, two key adaptor molecules in the TLR/NF-*κ*B pathway, trying to switch off the proinflammatory signal at the end of inflammatory response. While this mechanism efficiently regulates immune system responses [105], in senescent cells, its increase is insufficient to ameliorate the SASP [93, 106, 107]. As each miRNA has multiple targets, sustained and chronic expression of miR-146a-5p in senescent cells could affect many pathways other than the proinflammatory ones. In fact, mir-146a-5p has the mitochondrial encoded proteins ND1, ND2, ND4, ND5, and ND6, as well as ATP8 as putative and potential targets, suggesting that senescence may affect epigenetically the expression and function of complexes I and V. Of note, complex I is the most relevant one in determining ROS production in dysfunctional mitochondria [108]. In addition, miR-146a-5p can even target superoxide dismutase- (SOD-) 2, a major mitochondrial antioxidant enzyme [109] and Bcl-2, a known determinant of mitochondrial dynamics, involved in the regulation of mitochondrial fusion and fission [94]. Thus, it is easy to speculate that senescence-regulated miRNAs, which are transcribed with the goal of ameliorating inflammation, could also influence mitochondrial behavior, with a still unknown positive or negative effect on mitochondrial oxidative and energetic functions.

## 7. Conclusions and Future Prospects

**Figure 1:**
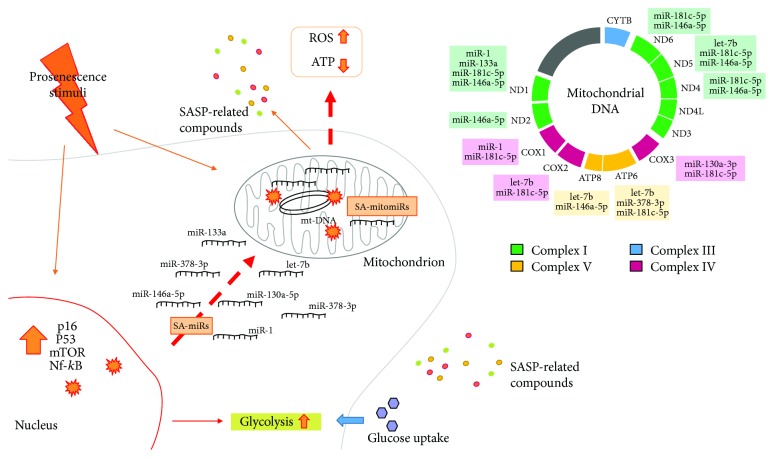
Potential effect of senescence-associated epigenetic rearrangement on mitochondrial function. Different damaging stimuli can induce senescence. Senescent cells bear peculiar metabolism and gene expression, which underlie a chronic proinflammatory program (SASP). This is accompanied by changes in the expression of a number of senescence-associated microRNAs (SA-miRNAs). Dysfunctional mitochondria play a major role in the promotion of the SASP. In turn, several SA-miRNAs can translocate to mitochondria (mitomiRs) and could target a plethora of mRNA with an important role within mitochondria, including transcripts derived from mitochondrial DNA (mtDNA). In this framework, we hypothesize a major role for SA-mitomiRs in determining the energetic, metabolic, and inflammatory status of senescent cells, mainly through their ability to regulate mtDNA-derived proteins.

Recent discoveries suggest that mitochondria are major determinants of aging. Senescent cells carry dysfunctional mitochondria, which are partly responsible for their proinflammatory program. Aged, dysfunctional, or damaged mitochondria can promote inflammaging even through continuous immune system stimulation. In the complex picture of the dynamic interaction between nucleus and mitochondria, microRNAs should be considered as new, relevant players. Despite the few data available on this specific topic, emerging evidence and bioinformatics studies indicate that miRNAs of nuclear origin profoundly affect mitochondrial function. This phenomenon seems to be of particular importance for the aging process, considering that many senescence-associated miRNAs have got or may have targets within mtDNA. Overall, a comprehensive view of the epigenetic and nonepigenetic mechanisms triggered by the senescence process is of importance to understand the overall phenotype of aged cells, which behave differently from a transcriptional, metabolic, energetic, and oxidative point of view (Figure 1). Such holistic view could eventually extend the spectrum of possibilities to target senescent cells and, more in general, the aging process, especially considering that miRNA-based, epigenetic therapies are already in progress in order to functionally reprogram target cells to a desired phenotype.

Cutting-edge technologies such as ultradeep sequencing and single-cell RNAseq will help to disentangle the importance of SA-mitomiRs in the aging process at both cellular and population level thus accumulating knowledge to better define dynamics underlying the epigenetic regulation of mitochondrial function in healthy aging and major ARDs.

## Figures and Tables

**Table 1:** List of miRs within human mitochondria.

Barrey et al. [78]		Bandiera et al. [20]	Sripada et al. [80]	Zhang et al. [60]	Mercer et al. [81]	Dasgupta et al. [82]
Human skeletal/muscular cells		HeLa cells	HeLa/HEK293	Skeletal muscle	143B cells	206 *ρ*° cells
let-7b	miR-193b	miR-328-5p	let-7b-5p	miR-1	miR-16	miR-181c
let-7g	miR-197	miR-494-3p	let-7g-5p		miR-146a	mR-146a
miR-19b	miR-199a-5p	miR-513a-5p	miR-107		miR-103	
miR-20a	miR-210	miR-638	miR-181a-5p			
miR-23a	miR-221	miR-1201	miR-221-5p			
miR-23b	miR-324-3p	miR-1246	miR-320a			
miR-24	miR-324-5p	miR-1275	miR-494-3p			
miR-34a	miR-365	miR-1908	miR-1275			
miR-92a	miR-423-3p	miR-1972	miR-1973			
miR-93	miR-484	miR-1973				
miR-103	miR-486-5p	miR-1974				
miR-106a	miR-490-3p	miR-1977				
miR-107	miR-503	miR-1798				
miR-125b	miR-501-3p					
miR-125a-5p	miR-532-3p					
miR-127-3p	miR-542-5p					
miR-133b	miR-574-3p					
miR-133a	miR-598					
miR-134	miR-720					
miR-149	miR-1974					
miR-151-5p	miR-1979					
miR-181a	miR-675^∗^					

**Table 2:** List of miRs within mouse and rat mitochondria.

Bian et al. [77]	Jagannathan et al. [83]					Kren et al. [76]	Das et al. [84]
Mouse liver	Mouse heart					Rat liver	Rat cardiomyocytes
let-7f-5p	let-7b	miR-151-3p	miR-1934-3p	miR-130a	miR-574-5p	miR-130a-3p	miR-181c
miR-101-5p	let-7a	miR-203-3p	miR-211-3p	miR-497	miR-148a-3p	miR-130b-3p	
miR-122-5p	let-7c	miR-212-3p	miR-3072-3p	miR-188-5p	miR-200c-3p	miR-140-5p	
miR-181b-5p	let-7f	miR-5112	miR-320-3p	miR-3098-5p	miR-300-3p	miR-320-3p	
miR-181d-5p	miR-149-3p	miR-135a-1-3p	miR-1199-5p	miR-30c-1-3p	miR-181b-5p	miR-494-3p	
miR-188-5p	miR-149-5p	miR-721	miR-5108	miR-712	miR-5131	miR-671	
miR-29a-3p	miR-23b	miR-125a-3p	miR-375-3p	miR-3102-5p			
miR-29c-3p	miR-1	miR-1904	miR-203-3p	miR-877-3p			
miR-361-5p	miR-29a	miR-1894-3p	miR-126-3p	miR-3963			
miR-432	miR-125b-5p	miR-3102-5p	miR-26a	miR-341-3p			
miR-494-3p	miR-29b	miR-494	miR-23a	miR-342-3p			
miR-680	miR-709	miR-1939	miR-27b	miR-423-3p			
miR-689	miR-22	miR-3470a	miR-99a	miR-3081-5p			
miR-690	miR-24	miR-144	miR-139-3p	miR-1895			
miR-705	miR-680	miR-3107	miR-378	miR-720			
miR-711	miR-21	miR-451	miR-27a	miR-1897-5p			
miR-721	miR-133a-3p	miR-1224	miR-29c	miR-3085-3p			
miR-720	miR-133a-5p	miR-2861	miR-30a	miR-3092			
miR-762	miR-133b	miR-2137	miR-30d	miR-2145			
miR-805	miR-128-3p	miR-1937c	miR-30e	miR-652-5p			
miR-671-5p	miR-3095-3p	miR-466i-5p	miR-3082-5p	miR-1187			
miR-1982-5p	miR-1937b	miR-705	miR-483-5p	miR-466h-3p			
