# Corrigendum to “Mitochondrial (Dys) Function in Inflammaging: Do MitomiRs Influence the Energetic, Oxidative, and Inflammatory Status of Senescent Cells?”

**DOI:** 10.1155/2019/8716351

**Published:** 2019-08-14

**Authors:** Angelica Giuliani, Francesco Prattichizzo, Luigina Micolucci, Antonio Ceriello, Antonio Domenico Procopio, Maria Rita Rippo

**Affiliations:** ^1^Department of Clinical and Molecular Sciences, DISCLIMO, Università Politecnica delle Marche, Ancona, Italy; ^2^IRCCS Multimedica, 20099 Sesto San Giovanni, Italy; ^3^Insititut d'Investigacions Biomèdiques August Pi i Sunyer (IDIBAPS), C/Rosselló 149-153, 08036 Barcelona, Spain; ^4^CIBER de Diabetes y Enfermedades Metabólicas Asociadas (CIBERDEM), Madrid, Spain; ^5^Center of Clinical Pathology and Innovative Therapy, Italian National Research Center on Aging (INRCA-IRCCS), Ancona, Italy

In the article titled “Mitochondrial (Dys) Function in Inflammaging: Do MitomiRs Influence the Energetic, Oxidative, and Inflammatory Status of Senescent Cells?” [1], there was an error in the affiliation details of the second author as he was wrongly listed as affiliated to affiliation number 3. The corrected authors' list and affiliations are shown above.
